# Glacial Indonesian Throughflow weakening across the Mid-Pleistocene Climatic Transition

**DOI:** 10.1038/s41598-019-53382-0

**Published:** 2019-11-18

**Authors:** Benjamin Petrick, Alfredo Martínez-García, Gerald Auer, Lars Reuning, Alexandra Auderset, Hanaa Deik, Hideko Takayanagi, David De Vleeschouwer, Yasufumi Iryu, Gerald H. Haug

**Affiliations:** 1 Max Planck Institute for Chemistry, Climate Geochemistry Department, Hahn-Meitner-Weg 1, 55128 Mainz, Germany; 2 Department of Biogeochemistry Frontier Bldg, 4F, Japan Agency for Marine-Earth Science and Technology (JAMSTEC) 2–15 Natsushima-cho, Yokosuka, Kanagawa 237-0061 Japan; 3 Kiel University, Institute for Geosciences, Ludewig-Meyn-Str. 10, 24118 Kiel, Germany; 4 RWTH Aachen University, Geological Institute, Wüllnerstrasse 2, 52062 Aachen, Germany; 5 Institute of Geology and Paleontology, Tohoku University, Aobayama, Sendai, 980-8578 Japan; 6 MARUM-Center for Marine and Environmental Sciences, Klagenfurterstraße 2-4, Bremen, 28359 Germany; 7 Geologisches Institut, Eidgenössische Technische Hochschule Zürich, 8092 Zürich, Switzerland

**Keywords:** Palaeoceanography, Palaeoclimate

## Abstract

The Indonesian Throughflow (ITF) controls the oceanic flux of heat and salt between the Pacific and Indian Oceans and therewith plays an important role in modulating the meridional overturning circulation and low latitude hydrological cycle. Here, we report new sea surface temperature and aridity records from the west coast of Australia (IODP Site U1460), which allow us to assess the sensitivity of the eastern Indian Ocean to the major reorganization of Earth’s climate that occurred during the Mid-Pleistocene Transition. Our records indicate glacial coolings at 1.55 and 0.65 million years ago that are best explained by a weakening of the ITF as a consequence of global sea level and tectonic changes. These coincide with the development of pronounced gradients in the carbon isotope composition of the different ocean basins and with substantial changes in regional aridity, suggesting that the restrictions of the ITF influenced both the evolution of global ocean circulation and the development of the modern hydrological cycle in Western Australia.

## Introduction

The Mid-Pleistocene Transition (MPT) represents a major transition in global climate that occurred between 1.25 and 0.6 million years (Ma) ago. The MPT is characterized by an intensification of ice ages and a change in the periodicity of the glacial/interglacial cycles from 41 ky to quasi-100 ky^1,2^. This change occurred in the absence of any significant variation in the orbital parameters that control Earth’s insolation^3,4^. The causes of this climatic transition are therefore likely related to Earth-internal mechanisms that modulate global climate response to astronomical forcing^4^. Numerous hypothesis to explain the MPT exist, but the ultimate cause of this shift in climate dynamics remains elusive^5–7^. Several studies have suggested that changes in the glacial mode of the Meridional Overturning Circulation (MOC) played a fundamental role in facilitating Northern Hemisphere ice sheet expansion during the MPT^8–11^.

The Indonesian Throughflow (ITF) constitutes the main pathway for the exchange of water, heat and salt between the Pacific and Indian Oceans, and therefore represents a key node in the return branch of the MOC^12^. In the modern ocean, the strength of the ITF modifies the heat and freshwater budgets and air–sea heat fluxes of the Pacific and Indian oceans on a seasonal basis, and influences the El Niño/Southern Oscillation (ENSO), the Indian Ocean Dipole (IOD), and the Asian monsoon^13,14^. Most climate model simulations predict a decrease in the ITF transport in response to anthropogenic warming, but the impacts of these changes on the MOC and global climate remain unclear^15,16^. In this context, the study of ITF dynamics and their impact on the eastern Indian Ocean (IO) during episodes of major climatic changes in the past can provide important insights on their sensitivity to different climate forcings.

On geological timescales, several studies have suggested that tectonically driven changes in the geometry and strength of the ITF occurring between 3 and 4 million years ago impacted the MOC and played a major role in the aridification of Africa, the shoaling of the global ocean thermocline, the onset of the Benguela upwelling, and ultimately in the intensification of Northern Hemisphere glaciations^17–22^. However, the sensitivity of the ITF to the major climate changes that occurred during the MPT, and their potential impact on the evolution of the MOC and global climate have, so far, not been investigated.

**Figure 1:**
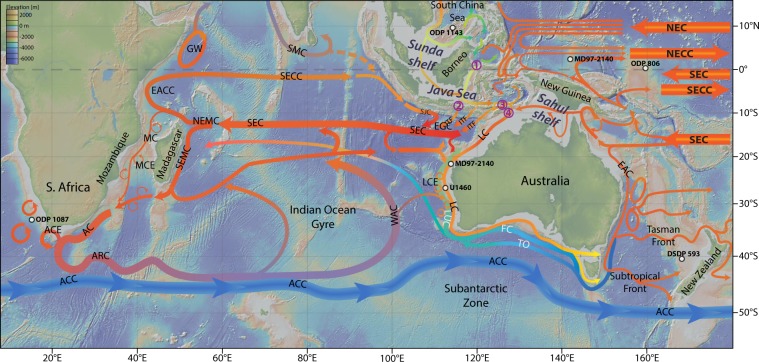
Overview map of oceanic circulation in the ITF region and its links to the Agulhas Current. Note the main path of the ITF through the Timor Strait feeding the Leeuwin Current (LC). Major oceanographic currents after^29,99–101^. The base map was generated with GeoMapApp (http://www.geomapapp.org)^102^. Other currents shown: The Indian and Pacific Ocean South Equatorial Current (SEC), South Equatorial Counter Current (SECC), and the Pacific North Equatorial (NEC) and North Equatorial Counter Current (NECC). The Northeast and Southeast Madagascar Current (NEMC and SEMC), the East Australian currant (EAC) East African Coastal Current (EACC), South Java Current (SJC), Mozambique Current (MC), Mozambique Current Eddies (MCE), Agulhas Return Current (ARC), Antarctic Circumpolar Current (ACC), West Australia Current (WAC), Leeuwin UnderCurrent (LUC), Leeuwin Current Eddies (LUE), Flinders Current (FC), Tasman Outflow (TO). Major ITF paths: 1) Makassar Strait; 2) Lombok Strait; 3) Ombai Strait; 4) Timor Passage.

The Leeuwin Current (LC) is a southward flowing current of low salinity, warm surface water, sourced from the ITF^23–26^ (Fig. 1). The current is driven by a steric height gradient, which pushes warm fresh water coming through the ITF down the west coast of Australia^26^. The temperature of the LC responds to changes in the strength and direction of the ITF^27–29^, and plays an important role in modulating the hydrology of western Australia^30^.

The recently drilled IODP Site U1460 (27°22.4867′S, 112°55.4265′E, 215 m water depth) is located in the eastern Indian Ocean, off of the west coast of Australia, at a location well suited to monitor changes in the strength of the LC and by extension the ITF in the past (Fig. 1). Here we report an orbital-scale reconstruction of SST changes at IODP Site U1460 over the past 2.5 Ma using the TEX_86_ paleothermometer (see methods section), which provides new insights on the variability of the ITF and its influence on the MOC across the MPT. In addition, we report an X-Ray fluorescence (XRF)-derived Ti/Ca record from the same site that allows us to infer past aridity changes in the region, and hence to evaluate potential links between regional aridity and the strength of the ITF.

## Results and Discussion

**Figure 2:**
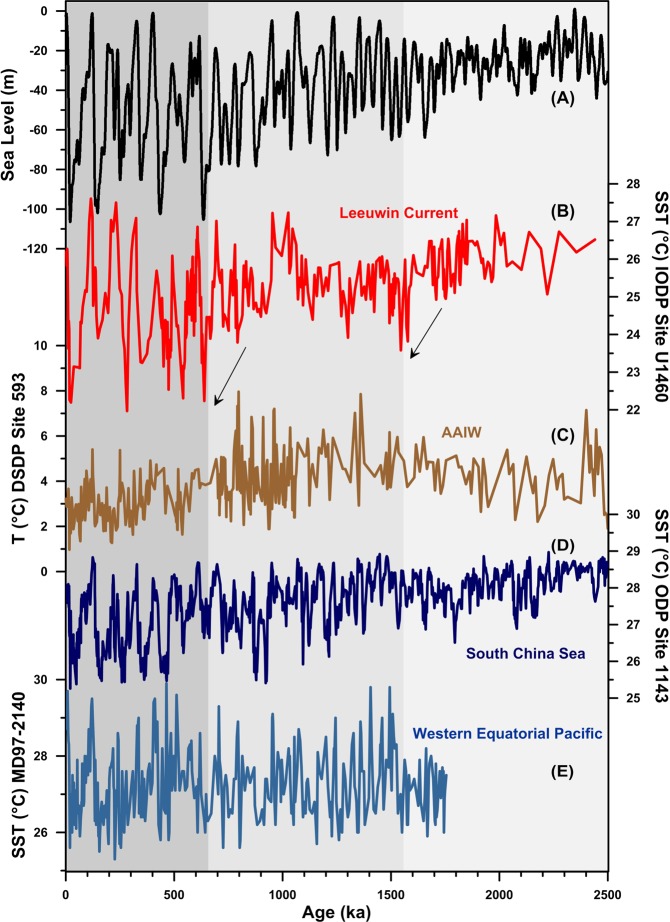
Sea level and sea surface temperature (SST) changes in the Leeuwin Current across the MPT. (**a**) Sea level reconstruction (black)^42^. (**b**) TEX_86_ SST estimates from IODP Site U1460 (red). (**c**) Antarctic Intermediate Water Temperature (AAIW) from DSDP 593^50^ (Brown). (**d**) South China Sea (ODP site 1143)^103^ and (**e**) Western Equatorial Pacific (MD97-2140) SST records^104^.

Between 2.5 and 1.55 Ma, annual mean SSTs oscillate between 27 °C during interglacials (G-IG) and 25 °C during glacials (Fig. 2). Around 1.55 Ma, there is a decrease in average glacial (to ~24 °C) and interglacial (to ~26 °C) temperatures, interrupted only by an interval of warming (up to 27 °C) around 1 Ma. At around 0.65 Ma interglacial temperatures increase to 27 °C, while glacial temperatures decrease to around 22 °C, marking a substantial increase in G-IG SST variability (Fig. 2). In summary, the changes around 1.55 Ma and 0.65 Ma represent an overall glacial cooling, while the changes at around 0.65 Ma are also characterized by an increase in glacial-interglacial variability. In the next sections we investigate the potential drivers of the observed temperature changes as well as their impacts on the evolution of the MOC and regional climate.

### Sensitivity of LC temperature to sea level changes across the MPT

**Figure 3:**
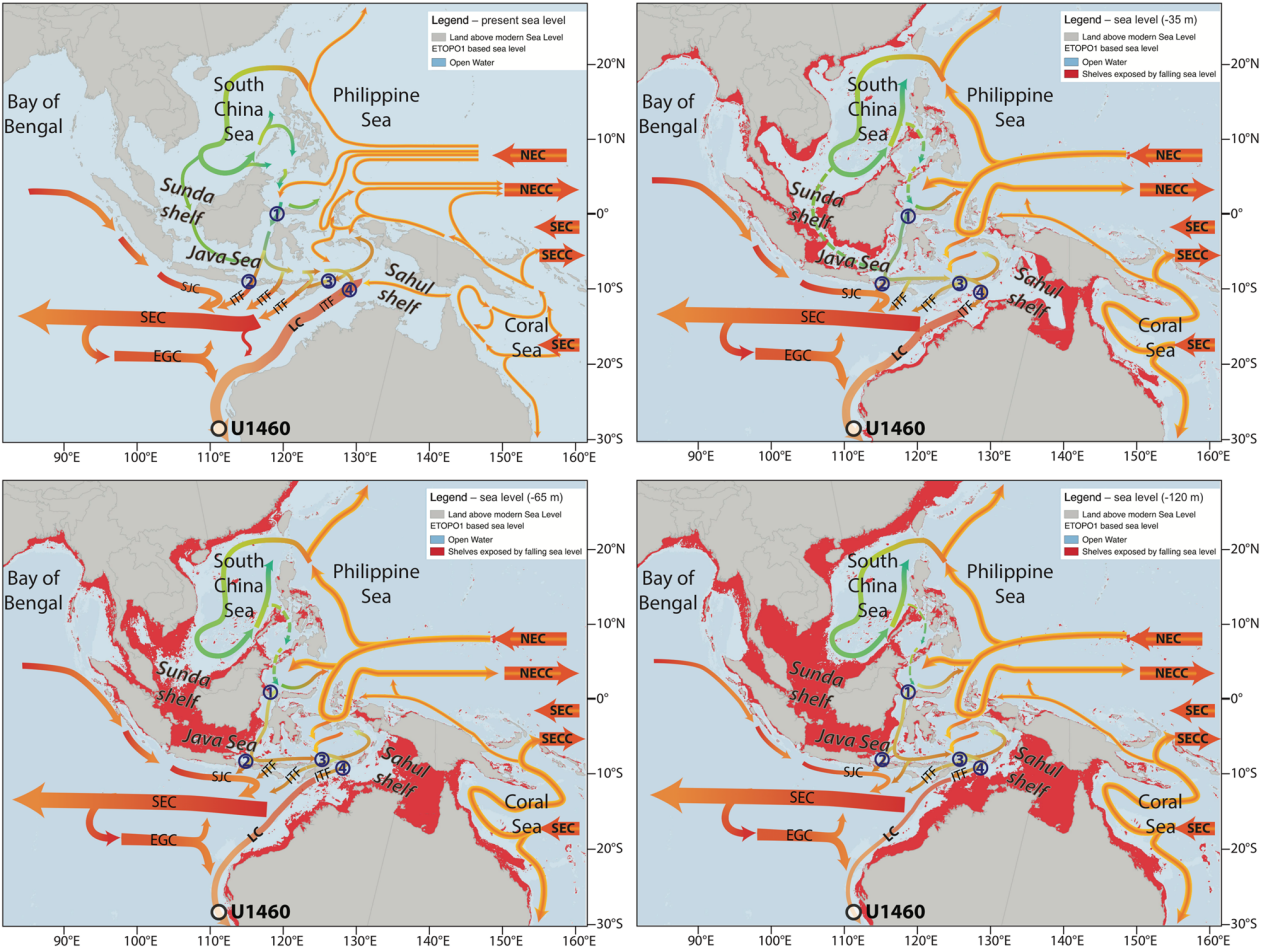
Effect of sea level changes on the geometry of the Indonesian Seas. Bathymetric reconstructions are based on the ETOPO1 (satellite measured sea level) reconstruction and the maps were made in QGIS^105^, and the estimated sea level drop during the different time periods is based on the reconstruction from^42^ (Fig. 2A). Major ITF paths: (1) Makassar Strait; (2) Lombok Strait; (3) Ombai Strait; (4) Timor Passage.

The ITF runs through an intricate network of channels and straits of variable depths and geometries that lay between the different islands of the Indonesian Archipelago (Fig. 1 & 3). The main water sources of the ITF are the North Pacific Ocean via the Mindanao Current and the South Pacific Ocean via the New Guinea Coastal Current^14^. Most of the ITF volume of water is transported through the Makassar Strait. Afterwards, part of its flow enters the Indian Ocean directly through the Lombok strait, but the bulk of the current is diverged eastward and exits the Indonesian seas through the Ombai and Timor straits^31^ (Fig. 1 and 3). South China Sea waters incorporated to the ITF via the Java and Sulu seas represent the main fresh water source and play an important role in the seasonal changes of ITF strength associated to the Asian monsoon^14^ (Fig. 3). Today, water transport through the ITF is governed by Godfrey’s Island rule^32,33^, which states that the number and shape of island exerts a major control on the shallow water movement through the system in relation to dominant wind fields^33^.

During the glacial periods of the Pleistocene some of the ITF channels were cut off and others substantially swallowed due to dropping sea levels as a result of increasing continental ice volume. The resulting exposure of shallow shelf areas in the Indonesian Archipelago and northwestern Australia significantly altered the sources and pathways of the ITF and the LC^34–37^, resulting in significantly different boundary conditions than those found in the modern ocean. Modeling studies show that the exposure of the Sunda and Sahul shelves in particular caused a major reorganization in the coupled ocean atmospheric system in the Indopacific region, which impacted the delicate balance of oceanographic conditions that control the southward flow of the LC^27,37^. In particular, a decrease in steric height gradients and increased wind shear both served to reduce LC strength as well as increasing upwelling along the Northwest shelf of Australia.

Model simulations of the effect of sea level changes during the last glacial maximum (LGM) also show that the restriction of the channels and the subsequent exposure of the continental shelf in Indonesia and Northern Australia results in cooling of the eastern equatorial Indian Ocean and reduction of LC strength, as well as an increase in salinity along western Australia^37^. These effects are supported by paleoceanographic reconstructions spanning the last 0.5 Ma based on foraminifera assemblages and stable isotope records showing a marked decrease in temperatures in the LC during the most recent glacial periods connected to sea-level driven constrictions of the ITF^38^. Therefore, it is possible that the cooling at 1.55 Ma and 0.65 Ma observed in our record were also linked to a weakening of the Leeuwin current due to a glacio-eustatic changes affecting LC source waters originating from the ITF.

Global sea level reconstructions across the MPT are subject to uncertainty, and the available records differ in the exact timing and amplitude of major sea level changes. We note that the timing of the reconstructed changes in SST in our record agrees better with sea level reconstructions based on the global benthic d18O stack^39^ than with individual records from the South Pacific^40^ and the Mediterranean Sea^41^. Most of the existing records show an average decrease in glacial sea level during the period from 1.7 to 1.4 Ma, ranging from 50 to 75 m^40–42^. Any change of more than 50 m would have significantly restricted the flow pathways of the ITF, both closing the link to the South China Sea through the Java Sea and restricting the flow through the Sulu Sea (Fig. 3C). These changes would have affected the strength of the ITF and the salinity and SST of the LC, which is consistent with the cooling observed around 1.55 Ma (Fig. 2B).

After 0.65 Ma, sea level reconstructions show an increase in the amplitude of glacial/interglacial variations, with larger glacial sea level drops of up to 120 m (Fig. 2A). The available sea level records differ on the exact timing of the switch, they place it either ~0.6 Ma^42,43^, or ~0.9 Ma^40,41^. A sea level drop from 65 m to 120 m would have completely exposed the Sunda and Sahul shelves eliminating the shallow flow paths of the ITF (Fig. 3D), and therefore significantly impacting the steric height difference that fuels the LC. These changes are consistent with the SST drop observed in our record at 0.65 Ma. In contrast to these glacial restrictions, some records also show an increase of up to 20 m in sea level during interglacial periods after 0.5 Ma, which could have opened up additional shallow passages within the Indonesian Archipelago hypothetically intensifying the LC, as well as increasing flow along the shallow shelf areas of the Australian Sahul shelf (e.g.^13^). These changes may explain the warmer interglacial temperatures observed in our record during the most recent interglacial periods.

**Figure 4:**
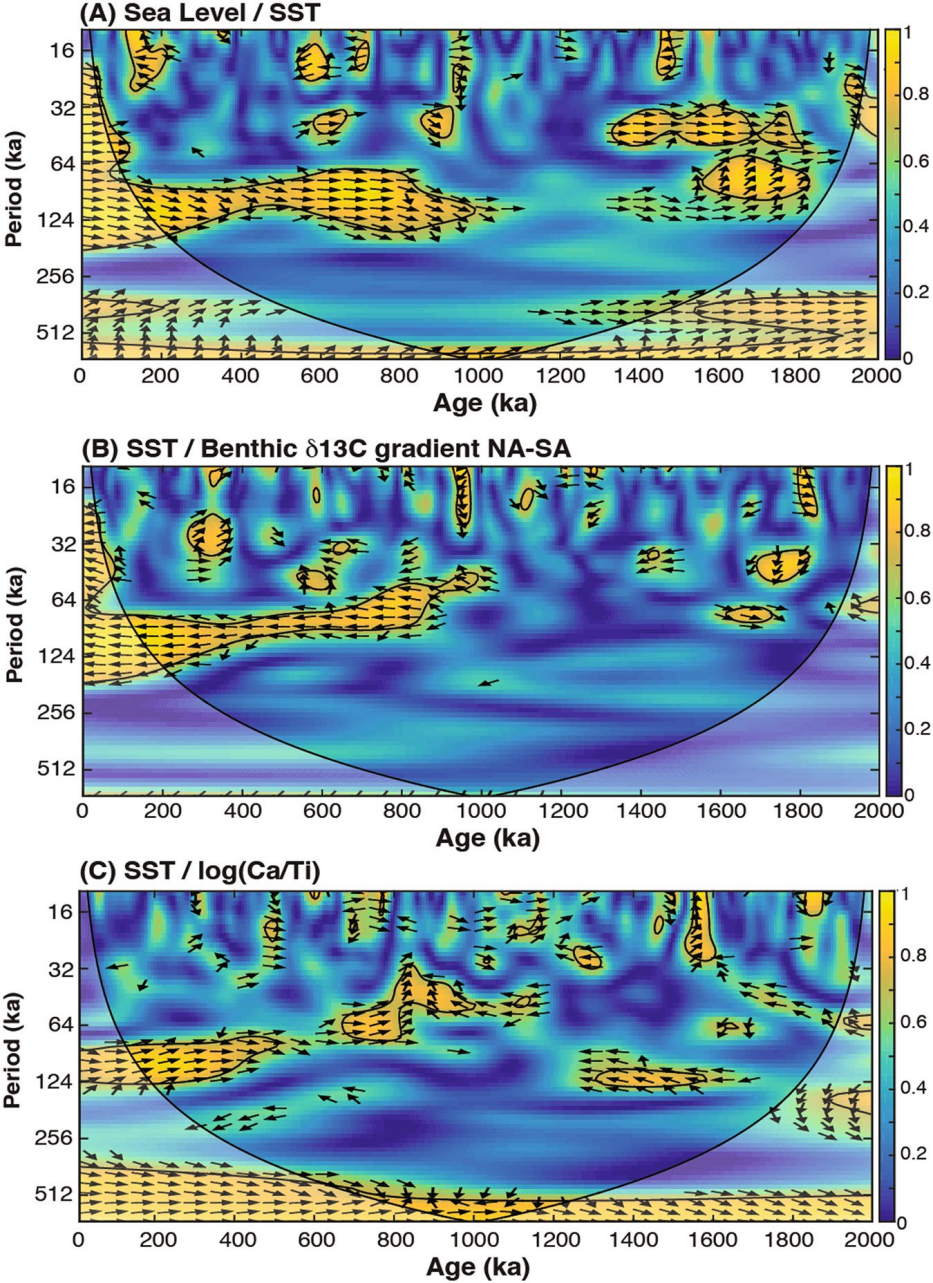
Cross-spectral analysis of climate records discussed in the text. (A) Sea-level and SST, (B) SST and benthic δ^13^C gradient, and (C) SST and Ti/Ca record. Squared wavelet coherence between two time series was computed using the methods proposed by^106^. The 95% confidence level against red noise was calculated using the Monte Carlo method and is shown as a thick contour that encloses the significant sections. The light shading indicates the region possibly influenced by edge effects. Black arrows indicate the phase relationship between the two time series.

The variance of the sea level and SST records increase at around 1.55 and 0.65 Ma, and a cross-spectral analysis of the global sea level record and the SST record from IODP Site1460 shows a strong coherence between the two records and a clear shift in the dominant frequency from 41ky to 100ky during the MPT (Fig. 4A). These observations support the hypothesis that the sea level drops and the concomitant increase in glacial/interglacial sea level variability after 1.7-1.4 Ma and 0.9-0.6 Ma would have resulted in a glacial weakening of the ITF that amplified the ITF G-IG oscillations, leading to the cooling and greater glacial-interglacial SST variability observed at IODP Site U1460.

In addition to sea level, tectonic changes in the Indonesian archipelago may have also contributed to the observed weakening of the ITF^44,45^. Several studies have shown that tectonic uplift has continued throughout the Pleistocene leading to a potential reconfiguration of the ITF passages^44,45^. Furthermore, they suggest an increase in the tectonic activity between 1.8-1.0 Ma which could have amplified the effect of SL in restricting channels and exposing more of the Sunda and Sahul shelves^46^.

Alternatively, changes in Sub-Antarctic Mode Water (SAMW) and Antarctic Intermediate Water (AAIW), incorporated in the Leeuwin Undercurrent (LUC) south of Australia, could have potentially affected the temperature evolution of the LC independently of sea level changes (Fig. 1). The LUC is a countercurrent flowing northward up the west coast of Australia beneath the LC^26,47^. The LUC is sourced from water masses subducted south of Australia and incorporates SAMW and AAIW^26,48^. It has been posited that, if the Leeuwin surface current were weakened due to changes in the ITF, the LUC could dominate along the coast^38,49^. Thus, the observed SST cooling at 1.55 Ma and at 0.65 Ma could have been influenced by changes in the temperature or the amount of AAIW transferred northwards via the LUC. However, the only existing record of AAIW temperature variability does not support this proposition, i.e. DSDP Site 593, located off New Zealand. This record shows similar glacial temperatures over the past 2.5 Ma, and warmer interglacial temperatures only between 0.9 and 1.4 Ma (Fig. 2C)^50^.

A decrease in the temperature of the source waters of the ITF linked to the global cooling caused by the Northern Hemisphere ice sheet expansion associated with sea level changes could potentially also explain the cooling trends observed in our record, independent of glacio-eustatic changes in the strength of the ITF. However, the available temperature reconstructions from the source areas of the ITF show stable glacial conditions in the Western Equatorial Pacific and no significant SST changes during the periods of major sea level drops at 1.55 and 0.65 Ma in the South China Sea (Fig. 2D).

These observations argue against a significant influence of changes in the temperature of AAIW and/or ITF source waters in the observed SST trends and suggest that the SST changes at IODP Site U1460 were driven by the effect of sea-level (and perhaps tectonic) changes on ITF dynamics.

### Potential impacts of ITF restrictions on the MOC

**Figure 5:**
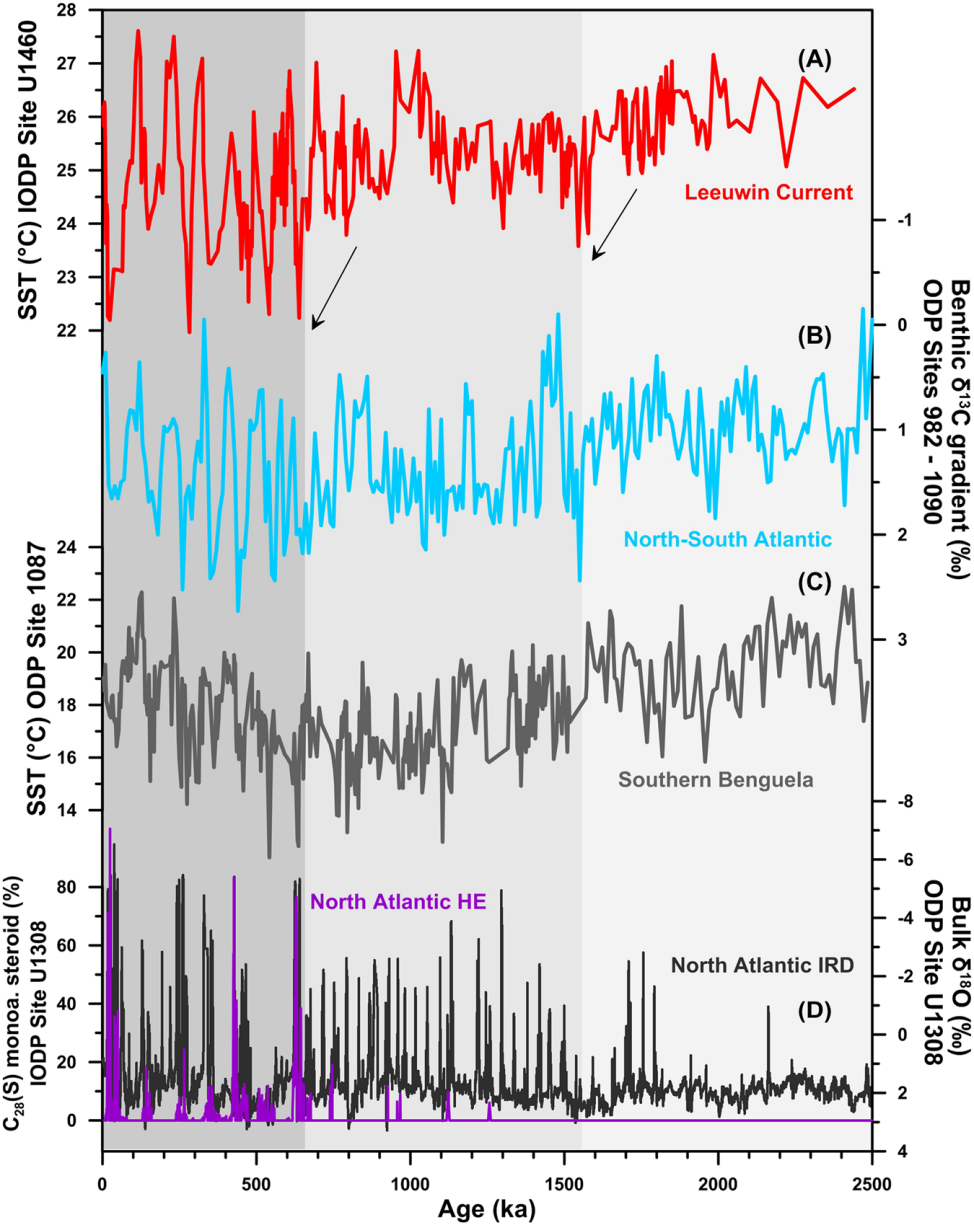
Links between changes in the ITF, the Agulhas Leakage and the MOC. (**a**) TEX_86_ SST estimates from IODP Site U1460 (red). (**b**) Benthic δ^13^C gradient between the South Atlantic (ODP Site 1090) and the Pacific (ODP 849)^9^ (Brown). (**c**) SST reconstruction from the SE Atlantic in the Agulhas leakage region^58^. (**d**) IRD and HE records from the North Atlantic (IODP Site U1308)^53,107,108^.

Past restrictions of the ITF in response to tectonically driven changes in the geometry of the Indonesian sea way are thought to have impacted the global thermohaline circulation during the early Pliocene^17,51,52^. However, the potential influence of ITF changes on the MOC during the MPT as a consequence of sea level changes has not been evaluated. Interestingly, the SST shifts observed in our record around 1.55 Ma and 0.65 Ma coincide with well-known changes the MOC recorded in the carbon isotopic composition (δ^13^C) of benthic foraminifera^9^ (Fig. 5). The available δ^13^C records show a major reorganization of deep water circulation at 1.55 Ma, which is characterized by an increase in δ^13^C gradients between the Pacific and Atlantic oceans, but also between the North and South Atlantic and the deep and intermediate South Atlantic^9^. At around 0.65 Ma benthic δ^13^C gradients tend to show an additional glacial increase in the interoceanic gradients, but also larger glacial/interglacial oscillations (Fig. 5B). In addition, at 0.64 Ma, sediment records from the North Atlantic indicate the onset of “Hudson Strait” Heinrich events (Fig. 5D), these are episodes of massive iceberg discharge associated with periods of Northern Hemisphere ice sheet instability that have been linked to weakening of the thermohaline circulation^53^.

The interpretation of the changes in benthic δ^13^C gradients is complex as they may have been influenced by several processes and feedbacks occurring along the MOC path during a period of major changes in Earth’s climate. For example, changes in the gradients between the North and South Atlantic can be caused by a decrease in the input of northern component water to the Southern Ocean and/or decreased exchange of CO_2_ between the atmosphere and surface water in Antarctic source areas^9^. Therefore, evaluating the exact impacts of the observed ITF glacial weakening on the evolution of the δ^13^C gradients and on the global oceanic meridional circulation is challenging and requires further modelling work. Nevertheless, the long-term trends and spectral characteristics of our SST record and the δ^13^C gradients are consistent with those expected if the ITF restrictions contributed to the observed glacial weakening of the MOC at 1.55 and 0.65 Ma (Fig. 5).

The ITF has a direct influence on the Agulhas Leakage, which funnels warm and saline water from the Indian Ocean into the South Atlantic Ocean that ultimately feeds the upper arm of the Atlantic meridional overturning circulation (Fig. 1)^54,55^. In the modern ocean, a strong ITF increases the Agulhas Leakage (AL) through the combined effect of an increase Agulhas Current transport (due to the additional input of water from the Pacific) and an increase in the number of Agulhas eddies^56^. In fact, some model results suggest that up to ~88% of the water carried by the ITF enters the Atlantic ocean via the AL after a 50 year period^31,57^.

On orbital timescales, the tectonically-driven decrease in the ITF from around 3.5 to 3 Ma has been hypothesized to cause a significant reduction in the AL that promoted the end of the persistent warm conditions across the Benguela region and facilitated the onset of upwelling in the Benguela system^20,52,58^. However, for other records located in the southern part of the Benguela system this connections have not been as clear^58,59^. Interestingly, the decrease in the ITF at 1.55 Ma coincides with the beginning of a cooling trend and an intensification of upwelling across the Benguela system (Fig. 5)^20,58^, suggesting that this sea-level driven restriction of the ITF was potentially linked to a decrease in the AL during this period. However, the start of the large amplitude glacial-interglacial SST oscillations observed in the ITF at around 0.65 Ma coincides with the onset of a glacial warming trend in the Agulhas region. This point marks a shift towards increased AL during the more recent glacial periods^58,60,61^ (Fig. 5C). This warming trend contrasts with the more stable glacial conditions observed in our record after 0.65 Ma. Benthic δ^13^C gradients tend to mirror the increase in G-IG variability observed in our record at 0.6 Ma, but they show a decrease in amplitude during the last glacial cycle. This benthic δ^13^C signal has been linked to the increased AL, evidenced by the SST warming trend found in the Agulhas region (Fig. 5)^58,60,61^. Collectively, these observations indicate a decoupling between the two systems during the last glacial cycle and suggest that changes in the MOC during this period may have been more affected by AL dynamics than by changes in the ITF. Longer records from the recent IODP Expedition 361 across the Agulhas region may allow a more detailed investigation of the links between the changes in the ITF and the evolution of the Agulhas current across the MPT.

### Effects of changes in the ITF on Australian climate

Today Australia is one of the most arid continents on the globe. Numerous studies have shown that present-day aridity has progressively developed in a series of steps starting around the Miocene period^62–64^. Most of the existing records of continental aridity spanning over the Plio-Pleistocene come from lakes that are currently dry or from dating the development of dunes or desserts^65–67^. While these records have provided invaluable insights on the development of aridity in Australia they are limited to the time windows covered by the archives analyzed and do not provide continuous climate records over the Pleistocene.

The ITF and its extension the LC are key in maintaining the relatively high coastal rainfall of Western Australia, creating a characteristic climate regime that has no counterpart to the hyper-arid coastal deserts found at similar latitudes in western South America and southern Africa^30^. In fact, model studies show that changes in the ITF can influence the rainfall of the entire Australian continent^34^. Models of ITF restriction during the Pliocene and LGM show that rainfall could be reduced up to 30% if the ITF is substantially reduced^34,37^. Recent studies from marine sediment cores show that two major changes towards more arid conditions at 3.6 and 2.4 Ma were likely linked to restrictions in the ITF^21,68,69^. IODP site 1460 provides an ideal opportunity to study the sensitivity of Australian climate to the major sea-level driven changes in the strength of the ITF identified in this study over the past 2 Ma.

**Figure 6:**
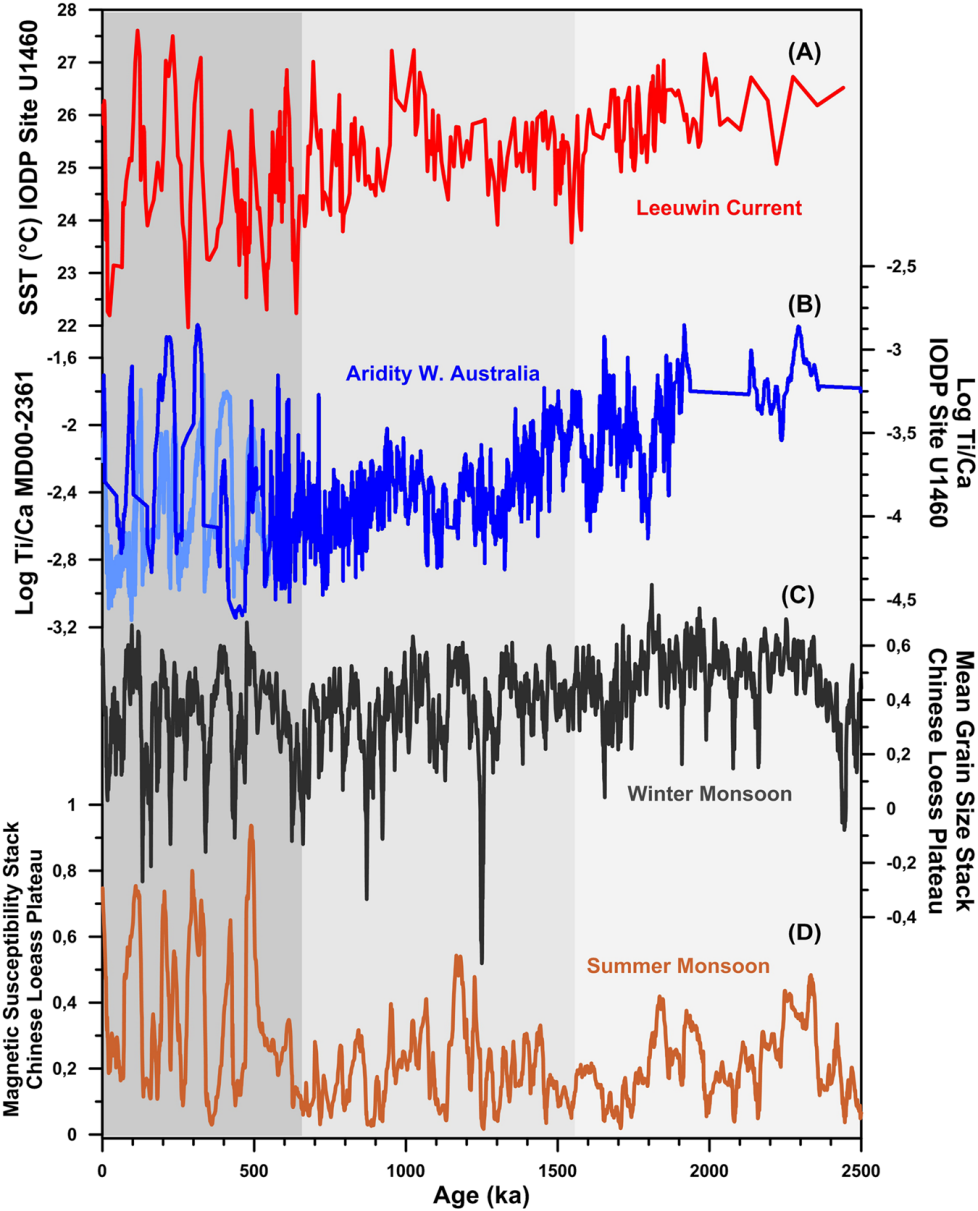
Links between ITF strength, aridity in north western Australia, and the Asian monsoon. (**a**) TEX_86_ SST estimates from IODP Site U1460 (red). (**b**) Records of rainfall based on Ti/Ca from IODP 1460 (this study) and MD00-2361^70^. (**c**,**d**) winter and summer monsoon reconstructions^82^.

To track changes in the hydrological cycle in our record, we use the Ti/Ca ratio as a tracer of terrestrial input (Fig. 6). Previous work shows that, just to the north of Site U1460, at Site MD00-2361, Ti/Ca preserves a good record of fluvial input, a parameter that is directly linked to the amount of rainfall in western Australia^70^ (Fig. 6B). This record shows a clear glacial-interglacial pattern with arid conditions during glacials and relatively humid climates during the interglacial stages over the past 0.5 Ma^70^. This scenario contrasts with that found in other records from the same latitude (20 S) offshore western South America and South Africa, which are characterized by humid glacials and dry interglacials^71^. This opposing patterns in Southern Hemisphere continental climate are thought to arise from the different sensitivity of the American, African and Australian continents to northward movements of the rain-bearing Southern Ocean westerlies during glacial stages^70^. In this respect, the absence/presence of a warm and strong LC during glacial/interglacial stages is thought to be key in explaining the characteristic arid/humid conditions of Western Australia over the past 0.5 Ma^38,72^.

Our new record from IODP Site U1460 is in good agreement with the existing reconstructions spanning over the late Pleistocene^70^, and suggests that the G-IG pattern characterized by large aridity/humidity changes started only around ~0.65 Ma ago coinciding with the increase in the G-IG SST variability in the IODP 1460 record (Fig. 6). Before 0.65 Ma, our Ti/Ca record indicates a long-term increase in continental aridity over the past 2.5 Ma, with a major aridification step starting at around 1.5 Ma. These changes match periods of increasing (and decreasing) aridity found in the rest of Australia. For example, an intensification of aridity at 1.5 Ma has been identified at Lake Bungunniain in southern Australia^73,74^, and a number of studies have reported final drying of saline lakes, expansion of dune fields, and expansion of deserts from 1.1 to 0.6 Ma^66,75–78^. Wetter conditions during more recent interglacials have been identified in lakes and dune deposits from both western and southern Australia^63,79–81^. These changes may be partially influenced by the global cooling that occurred over the same period and/or by changes in the strength of the Asian monsoon (see next section), but our data suggest that changes in ITF strength likely played an important role.

### Feedbacks between the ITF and the Asian monsoon

In the modern ocean the Asian monsoon drives the hydrological cycle in the Indonesian Seas and influences both the strength of the ITF and the seasonal wind and precipitation patterns in Northern Australia. During boreal winter northwesterly winds travelling across the Indonesian Seas favor the transport of humid air towards the northern coast of Australia, but rain-bearing monsoonal wind is often kept offshore limiting its impact on the northern coast of Australia. During boreal summer the wind pattern reverses and southeasterly winds cause a general decrease in precipitation. Thus, the Australian monsoon is typical in the sense that it shows characteristic seasonal changes in wind directions and precipitation, but its persistence and frequency during the summer months is more limited than in regions located further north, e.g. Indonesia and South East Asia.

These monsoonal changes also have a large effect on ocean circulation. During boreal winter the north westerly monsoon winds push low-salinity Java Sea surface water into the southern part of the Makassar Strait, creating a ‘freshwater plug’ that inhibits the warm surface water from the Pacific Ocean from flowing southward into the Indian Ocean^13^. This leads to a cooler Indian Ocean surface, which in turn weakens the Asian monsoon. This pattern reverses in boreal summer when south easterly winds move more-saline Banda Sea surface water into the southern Makassar Strait leading to a stronger ITF, which intensifies the Asian monsoon^13^.

In this context a weakening of the ITF during the periods of sea level change identified in this study at 1.55 Ma and 0.65 Ma could have acted as a negative feedback on the intensity of the Asian monsoon. Alternatively, an intensification of the winter monsoon and/or a weakening of the summer monsoon circulation during this time periods may have weakened the ITF and contributed to increase aridity in Australia. The available monsoon records from the Chinese Loess Plateau show a general increase in the intensity of the winter monsoon during glacial stages over the past 2.5 Ma, but the timing and pattern of these records show little correspondence with those reconstructed here in the ITF (Fig. 6C)^82^. The summer monsoon records are characterized by stable glacial conditions, but show a significant increase in intensity during the interglacial periods after around 0.5 Ma (Fig. 6D). These changes tend to coincide with warmer interglacial SST in our record and suggest the existence of a positive feedback between the strength of the ITF and the intensity summer monsoon during late Pleistocene interglacials. Interestingly, this change towards stronger interglacial summer monsoon and a more vigorous ITF roughly coincides with the appearance of more humid interglacial periods in our Ti/Ca record, suggesting that the ITF/monsoon feedback could have played a role in the development of the modern hydrological cycle in western Australia.

## Conclusions

Our study evidences of a number of significant changes in the strength of the ITF occurred during the MPT. The new data reported here indicates that that glacio-eustatic ITF restriction potentially coupled with tectonic changes caused a significant cooling in the Indian Ocean at 1.55 and 0.65 Ma. These changes coincide with the development of stronger interoceanic benthic δ^13^C gradients suggesting that the restriction of the ITF during the MPT influenced the evolution of global circulation at both 0.65 and 1.55 Ma. Our data also shows a long term glacial aridification trend in western Australia starting with the first restriction of the ITF at around 1.55 Ma. The development of stronger ITF G-IG variability after 0.65 Ma coincides with more intense interglacial Asian summer monsoon and increased rainfall in Western Australia. These observations suggest the establishment of interglacial oceanic and atmospheric circulation patterns similar to those observed in the region today at the end of the MPT.

Modern observational evidence indicates that the strength of the ITF is impacted by interannual and multidecadal climate variability. Historical data and model simulations suggest a reduction of the ITF in response to global warming, but it remains unclear how the associated changes in the Indian Ocean might impact the downstream Agulhas Current system and eventually the Atlantic Meridional Overturning Circulation^31^. Although, the timescales, boundary conditions and drivers of ITF restrictions are significantly different, the data presented here indicates that long-term changes in the strength of the ITF can have a significant impact on global ocean circulation and regional climate.

## Methods

### Age model

In order to create the age model for IODP site U1460, first we used the initial IODP shipboard bio-stratigraphic age model^83,84^ (Fig. S1). Then we used the high-resolution SST to adjust IODP Site U1460 to the LR04 chronology. We note that age model uncertainty may arise from aligning the SST record to LR04^85^, which assumes that there is no lead or lag of the SST record compare to the benthic oxygen isotopic record. The LR04 chronology imparts a lag of 5 ka compared to orbital forcing^85^. The SST data at Site U1460 thus may contain an error of up to 5 ka in the timing of the start or end of glacials and interglacials. However, this uncertainty does not affect any of the conclusions of the paper.

### XRF scanner analysis

Elemental data were collected using an XRF Core Scanner II (AVAATECH Serial No. 2). The data reported here have been acquired by a Canberra X-PIPS Silicon Drift Detector (SDD; Model SXD 15C-150–500) with 150 eV X-ray resolution, the Canberra Digital Spectrum Analyzer DAS 1000, and an Oxford Instruments 50 W XTF5011 X-Ray tube with rhodium (Rh) target material. Elements were collected at a resolution of 2-cm down-core, over a 2 cm^2^ area with down-core slit size of 10 mm, using generator settings of 10 kV, a current of 0.15 mA, and a sampling time of 20 seconds directly at the split core surface of the archive half. The split core surface was covered with a 4-micron thin SPEXCerti Prep Ultralene1 foil to avoid contamination of the XRF measurement unit and desiccation of the sediment. Raw data were processed by analysis of X-ray spectra using the Iterative Least square software (WIN AXIL) package from Canberra Eurisys. A number of sections were unable to be scanned due to being composed of unsuitable material (gravel) or were too irregular to yield reliable data. This resulted in a lower resolution XRF data in the upper part of the core.

### Biomarker analysis

The sediment samples were freeze-dried, crushed and extracted using an ASE 350 extraction system. We used a new method developed at the Max Plank Institute for Chemistry (MPIC) that allows the separation into different polarity fractions simultaneously to the extraction process (Auderset and Martínez-García, *in review*). The polar fraction was dried down in a roto-evaporative system and filtered through a 20 μm filter using a 1.5% IPA/Hexane mixture. The samples were then placed under a nitrogen stream until dried. They were then diluted in 300 ml of 1.5% IPA/Hexane and analyzed using an HPLC-MS system following the methods described by Hopmans *et al*.^86^. Standards and blanks were run for every batch of 20 samples in order to track reproducibility and cross contamination.

### Considerations about the TEX_86_ paleotemperature index

The TEX86 paleo-temperature proxy has been used successfully in a wide number of environments and timescales to reconstruct past SSTs^87^. There are a number of calibrations for converting TEX_86_ values to SSTs. We tested the two main formulas for calculating SSTs from TEX_86_: the logarithmic TEX_86_^H^ calibration^88^ and the spatially-varying TEX_86_ BAYSPAR calibration^89^. Both TEX_86_ calibrations showed the same trends, and only minor differences in the absolute temperature values.

A number of indexes, described below, have been developed in recent years to evaluate any potential non-thermal influence on the reconstructed SST (Fig. S2). The BIT index is used to track the amount of terrestrial GDGT input at a site^90,91^. IODP site 1460 has average BIT values of 0.06 similar to the values of around 0.1 found in open ocean waters^90^, suggesting minimal contribution from terrestrial GDGTs. This is in good agreement with the low Ti concentrations, which suggest that terrestrial material represents a small percentage of the overall sedimentary content. The Methane Index (MI) is used to exclude any data affected by gas-hydrate-related anaerobic oxidation of methane^92^. At IODP site 1460, the average MI values were 0.22, and all the data fell below the recommended 0.50 threshold value (Fig. S2). In addition to the MI we used the GDGT-0% index to evaluate the amount of sedimentary archaeal methanogenesis^93,94^. For IODP site 1460, the average value for this index was 29%, and all of the samples fell far below the 67% recommended threshold (Fig. S2). To test whether our sediments have unusual distributions like those found in the Red Sea, we used the GDGT_RS_ %. The average was 12%, and, once again, all the samples were well below the limit for rejection of 24%^95^ (Fig. S2). The ring index is used to evaluate whether the TEX_86_ is being produced are influenced by non‐thermal factors and/or deviate from modern analogues^96^. Samples are rejected if they are more than 0.3 away from the Ring Index – TEX86 correlation derived from core top data. For this study only three data points fall outside of this error envelope and are not far outside the envelope. Therefore, we have decided not to remove them (Fig. S2). Finally, the GDGT [2/3] ratios can be used to assess the impact of changes in the depth at which the GDGTs have been produced through time^97,98^. The [2/3] ratios are low during the entire interval, as expected from GDGTs that have been produced at the surface.

## Supplementary information

Supplementary info
