# Supplementary material for: Glacial Indonesian Throughflow weakening across the Mid-Pleistocene Climatic Transition
Source: Sci Rep. 2019 Nov 18;9:16995. doi: 10.1038/s41598-019-53382-0 (PMC6861309; doi:10.1038/s41598-019-53382-0)
Supplement: Supplementary file 1 — Supplementary info [file 41598_2019_53382_MOESM1_ESM.docx]

**Glacial Indonesian Throughflow weakening across the Mid-Pleistocene Climatic Transition**

Benjamin Petrick, Alfredo Martínez-García, Gerald Auer, Lars Reuning, Alexandra Auderset, Hanaa Deik, Hideko Takayanagi, David De Vleeschouwer, Yasufumi Iryu, Gerald H. Haug

**Supplemental Information**

**
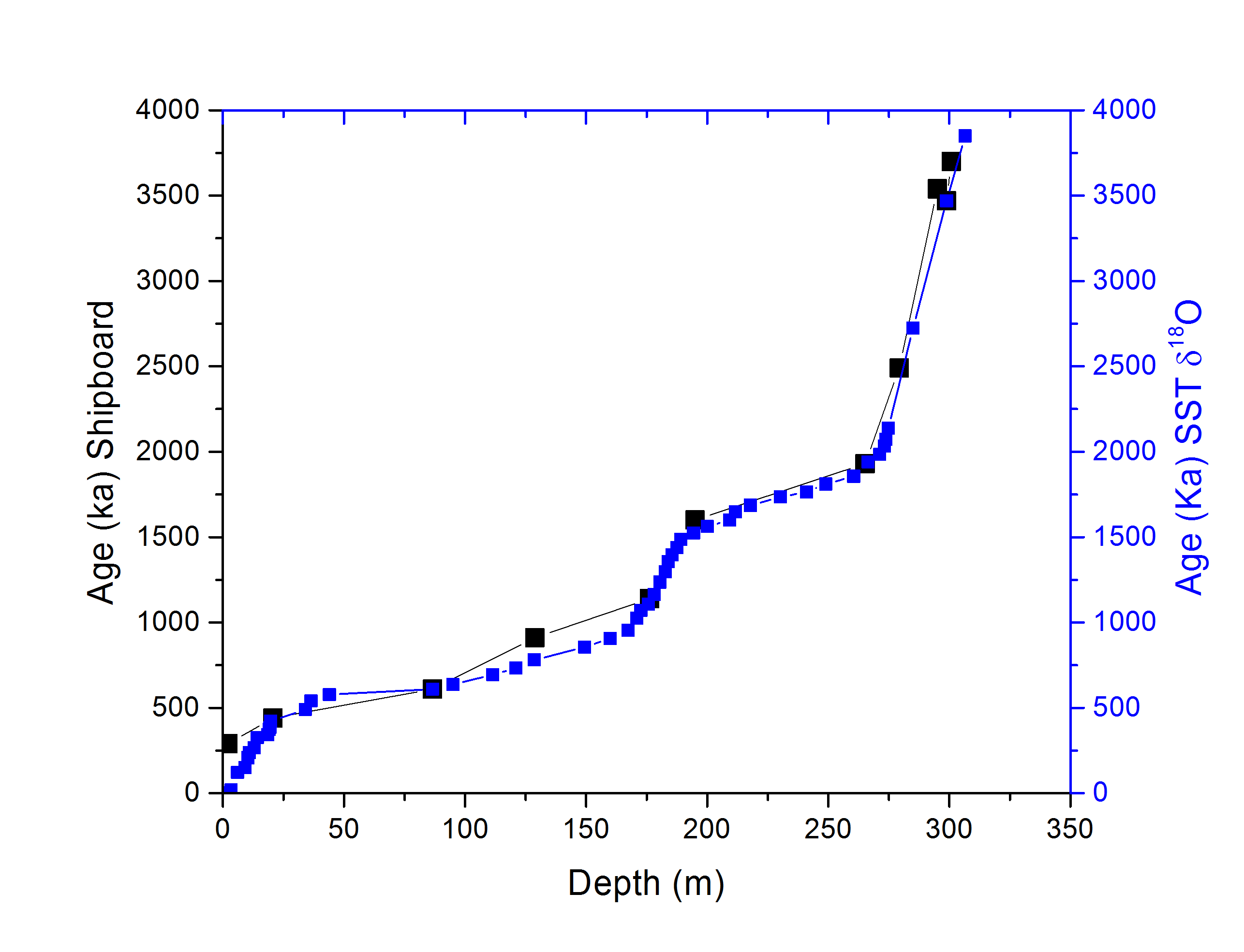
**

**Figure S1**. Initial shipboard biostratigraphy age model (black) compared to the new age model (blue) developed for the IODP 1460 in this study based on the alignment of SST and LR04 δ^18^O stack with tie points indicated.

**
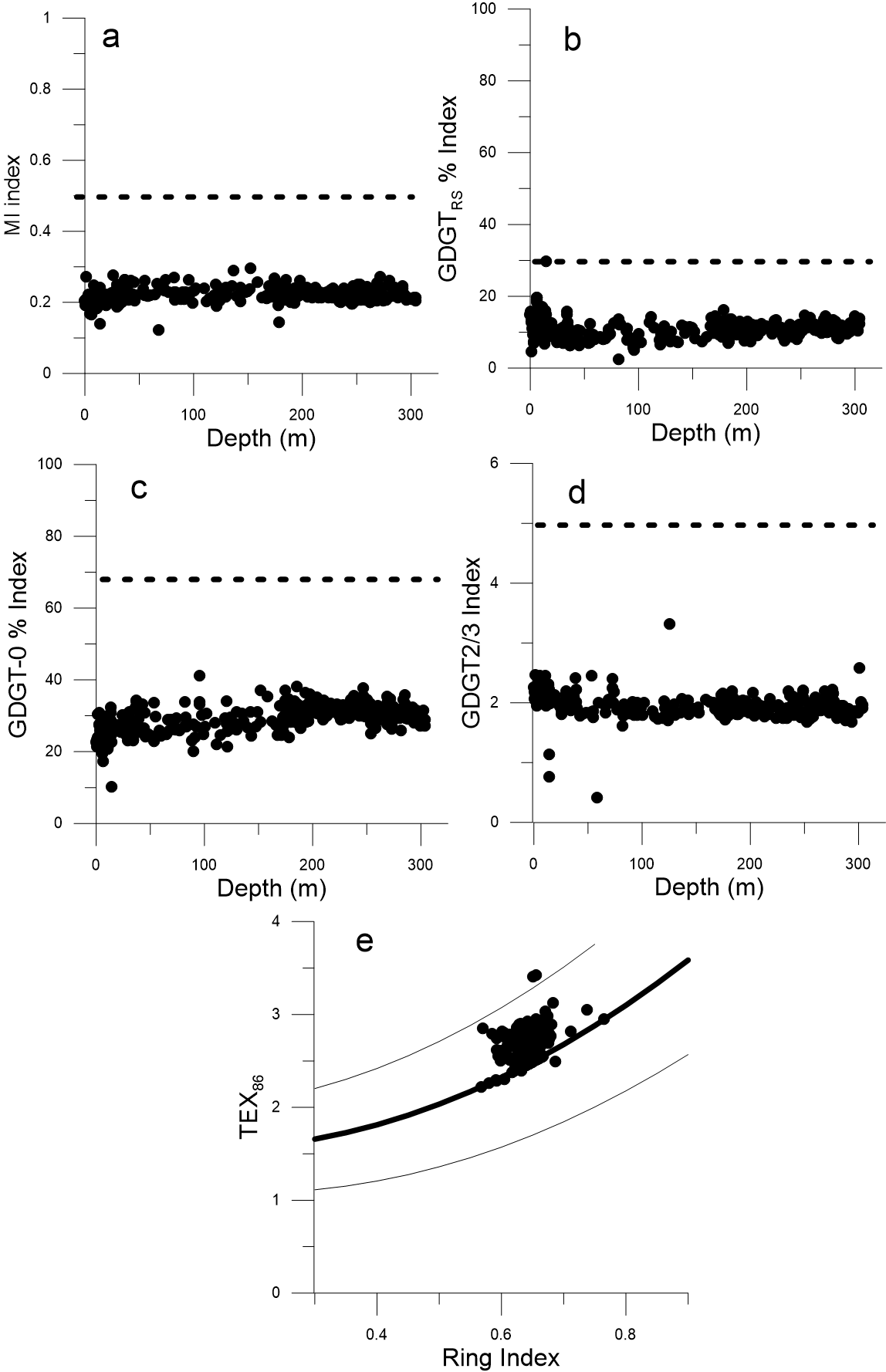
**

**Figure S2**. Test of potential non-thermal influences on the TEX_86_ SST record referred to in the text and their recommended thresholds shown by the doted lines. Note all the data is on the depth scale. a) the MI index b) GDGT_RS_ % c) GDGT-0 % d) GDGT2/3 e) Ring Index. All the data fall below the recommended values for these indexes, suggesting negligible non-thermal influences on the reconstructed SST.
